# Risk of Cancer in Patients With Crohn’s Disease 30 Years After Diagnosis (the IBSEN Study)

**DOI:** 10.1093/crocol/otad057

**Published:** 2023-10-03

**Authors:** Benoit Follin-Arbelet, Milada Cvancarova Småstuen, Øistein Hovde, Lars-Petter Jelsness-Jørgensen, Bjørn Moum

**Affiliations:** Institute of Clinical Medicine, University of Oslo, Oslo, Norway; Department of Gastroenterology, Oslo University Hospital, Oslo, Norway; Østfold University College, Halden, Norway; Institute of Clinical Medicine, University of Oslo, Oslo, Norway; Department of Public Health Oslo Metropolitan University, Oslo, Norway; Institute of Clinical Medicine, University of Oslo, Oslo, Norway; Innlandet Hospital Trust, Gjøvik, Norway; Østfold University College, Halden, Norway; Østfold Hospital Trust, Sarpsborg, Norway; Institute of Clinical Medicine, University of Oslo, Oslo, Norway; Østfold Hospital Trust, Sarpsborg, Norway

**Keywords:** Cancer, Crohn’s disease

## Abstract

**Background:**

Patients with Crohn’s disease (CD) are most often diagnosed as young adults; therefore, long-term studies are needed to assess the risk of cancer over their lifetime. Thus, the aims of the present study were to determine the risk of cancer in a Norwegian population-based cohort (the Inflammatory Bowel South Eastern Norway [IBSEN] study), 30 years after diagnosis, and to assess whether patients with CD were at an increased risk of specific cancer types.

**Methods:**

The IBSEN cohort prospectively included all incident patients diagnosed between 1990 and 1993. Data on cancer incidence were obtained from the Cancer Registry of Norway. Overall and cancer-specific hazard ratios (HRs) for CD patients compared with age- and sex-matched controls were modeled using Cox regression. Standardized incidence ratios (SIRs) were estimated compared to the general population.

**Results:**

In total, the cohort included 237 patients with CD, and 36 of them were diagnosed with cancer. Compared to the general Norwegian population, patients with CD had an increased overall risk of cancer (HR = 1.56, 95% CI: 1.06–2.28), particularly male patients (HR = 1.85, 95% CI: 1.08–3.16). The incidence of lung cancer and nonmelanoma skin cancer was increased; however, the difference was not statistically significant (SIR = 2.29, 95% CI: 0.92–4.27 and SIR = 2.45, 95% CI: 0.67–5.37, respectively).

**Conclusions:**

After 30 years of follow-up, the risk of all cancers in patients with CD was increased compared to the general population.

## Introduction

Crohn’s disease (CD) is a chronic inflammatory disease of the gastrointestinal tract, and complications associated with longstanding inflammation might arise after several decades.^1^ The disease course of CD is heterogeneous; while some patients have a mild disease course, a large proportion of patients require surgical interventions. Moreover, some patients may suffer serious extraintestinal manifestations involving joints, skin, eyes, and even malignancies.^2–4^ Additionally, side effects of medical treatments including immunosuppressive drugs raise the risk of distinct extraintestinal cancers, such as lymphoma and nonmelanoma skin cancer (NMSC).^3,4^

Longitudinal population-based studies are best suited to represent the spectrum of CD phenotypes and are therefore to assess the long-term disease course and prognoses of patients with CD.

The Inflammatory Bowel South Eastern Norway (IBSEN) study found that patients with CD were not at an increased risk for cancer compared to controls at 20 years follow-up.^5^

The aims of the present study were to determine the risk of cancer in the IBSEN study, 30 years after diagnosis, and to assess whether patients with CD were at an increased cancer-specific risk, compared with the general Norwegian population.

## Material and Methods

### Patient Population

The IBSEN study prospectively included all patients with newly diagnosed inflammatory bowel disease (IBD) residing in 4 counties in South-Eastern Norway from January 1, 1990, to December 31, 1993. All patients were invited to participate in prescheduled follow-up visits and examinations at 1, 5, 10, and 20 (±1) years after enrollment. Medical and surgical treatments were recorded until the last prescheduled visit. The diagnosis was revised for up to 10 years after enrollment. Based on the clinical information collected at the time of diagnosis, the disease phenotype was retrospectively classified according to its location and behavior. The design, methods, and procedures have previously been described in detail elsewhere.^6,7^

### Data Sources and Definitions

All Norwegian residents are assigned a unique digital identification number which allows for the linking of data between different registries. The cancer incidence and mortality data were obtained from the Cancer Registry of Norway (CRN). All medical doctors in Norway are obliged to report new cancer cases to the CRN. Data from the CRN were coded according to the International Classification of Disease (ICD-10).^8^

Cancer cases with a low certainty of diagnosis were not included. Neoplasms of uncertain behavior (ICD-10 D codes) were not included either.

Each patient in the IBSEN cohort was matched for age and sex with 5 individuals residing in the same geographical area at the time of diagnosis, randomly drawn from the Norwegian National Population Register.

Prescription of thiopurines or TNF-α inhibitors is defined as the presence of any recorded prescription, up to 20 years after the diagnosis.

### Statistical Analysis

Categorical data are presented as counts and percentages.

Event was defined as the first occurrence of any cancer from the date of enrollment until December 31, 2020, or the end of follow-up. Follow-up started from the date of enrolment and ended at the time of emigration, end of study, or death.

Prescription of thiopurine or biologics was defined by the presence of any prescription record at any time, until the last scheduled visit at 20 (±1) years.

The cumulative incidence of the first cancer occurrence was plotted using the Kaplan–Meier method adjusted for competing risks. Overall cumulative mortality was plotted using the Kaplan–Meier method.

Cancer risks for patients compared to controls were modeled using the Cox proportional hazard model stratified by matched case–control sets. When the analyses were restricted to patients only, the regression models were adjusted for age and sex. The results are expressed as hazard risk ratios (HRs) with 95% confidence intervals (CIs).

Standardized incidence ratios (SIRs) were computed as the ratio between the observed and expected numbers of incident cancers. Expected incident cancer estimations were derived from openly available incidence data from the CRN for the corresponding sex, time, regions, and age groups.^9^ The incidence of “cholangiocarcinoma” (ICD-10 C22.1) and “overlapping cancer of the anus and rectum” (ICD-10 C21.8) was not available from this data source. Therefore, the incidence of these cancer subtypes contributes to the SIRs of liver cancer and anal cancer, respectively.

Confidence intervals for the SIRs were calculated using the Wilson and Hilferty approximation.

*P*-values less than .05 were considered statistically significant. All analyses were considered exploratory; no correction for multiple testing was done.

All analyses were performed using R statistical software version 4.2.1 and the survival package version 3.3.

### Ethical Considerations

The study has been approved by the Regional Committee for Medical Research Ethics and the University Hospital Data Protection Officer.

The interpretation and reporting of these data are the sole responsibility of the authors, and no endorsement by the CRN is intended, nor should it be inferred.

## Results

In total, 237 patients were included, and 3 were lost to follow-up due to emigration. At diagnosis, disease location in the colon only was the most common, with 48.5% of patients, and the most common behavior was nonstricturing nonpenetrating (62%). The median age of patients alive at the end of the study was 55 years old. The cohort characteristics are presented in Table 1.

**Table 1.** Demographic and clinical characteristics of the IBSEN cohort.

	Number of patients (%)	Number with incident cancer (%)
Females	118 (49.8)	17 (47.3)
Males	119 (50.2)	19 (52.7)
Age at diagnosis		
Age <17	29 (12.2.)	3 (1.3)
Age 17–39	138 (58.2)	12 (5.1)
Age ≥40	70 (29.5)	21 (8.9)
Location at diagnosis		
Ileal	64 (27.0)	10 (4.2)
Colonic	115 (48.5)	17 (7.3)
Ileocolonic	54 (22.8)	9 (3.8)
Upper disease	4 (1.7)	0 (0)
Disease behavior at diagnosis		
Non-stricturing non-penetrating	147 (62.0)	20 (8.4)
Stricturing	64 (27.0)	11 (4.6)
Penetrating	26 (11.0)	5 (2.1)
Smoker at diagnosis	95 (40.1)	16 (6.8)
Prescription until 20 years of follow-up		
Thiopurines	95 (40.1)	15 (6.3)
TNF-α inhibitors	42 (17.7)	9 (3.8)

**Figure 1.**
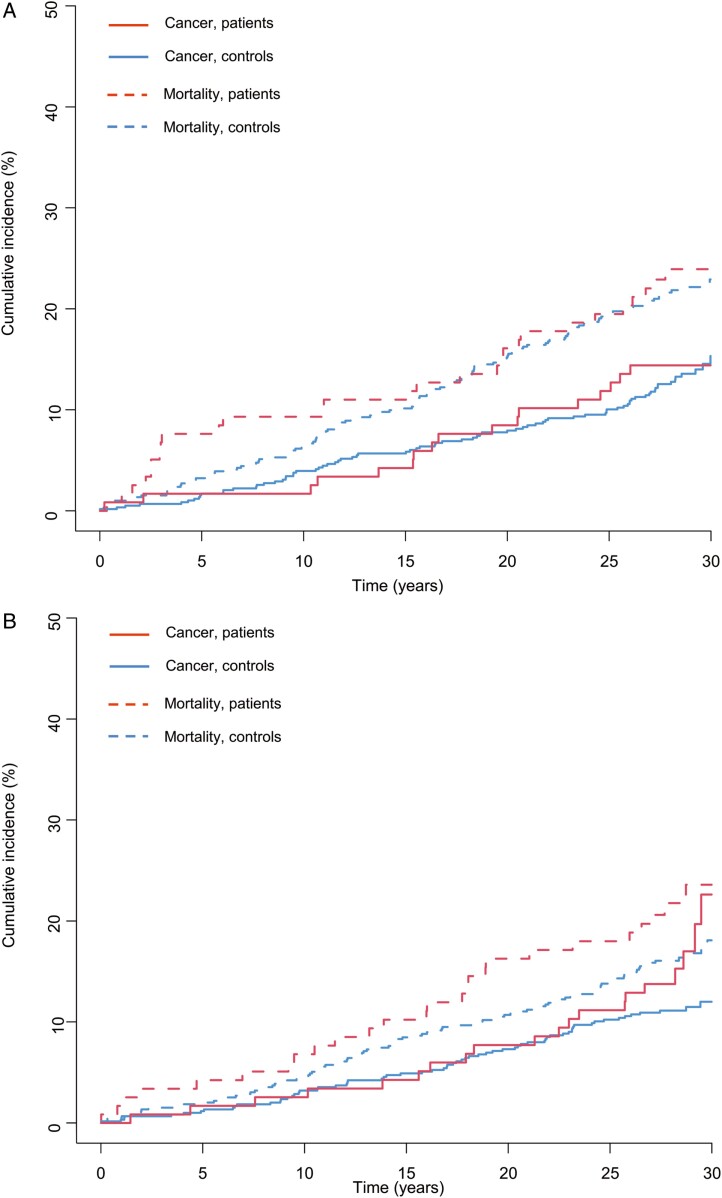
A, Cumulative incidence of cancer and death in female patients and controls. B, Cumulative incidence of cancer and death in male patients and controls.

Cancer was diagnosed in 36 patients, and 1 patient was diagnosed with cancer twice. Figure 1 illustrates the cumulative incidence of cancer and death stratified by gender.

Patients with CD were almost 1.6 times more likely to be diagnosed with cancer, compared to their matched controls (HR = 1.56, 95% CI: 1.06–2.28) (Table 2). Male patients specifically were at a significantly higher risk of being diagnosed with cancer (HR = 1.85, 95% CI: 1.08–3.16), while the cancer risk in female patients was not statistically significantly different from their controls (HR = 1.32, 95% CI: 0.76–2.28).

**Table 2.** Hazard ratios for occurrence of first cancer in IBSEN patients.

	Hazard ratio	95% CI
All patients	**1.56**	**1.06–2.28**
Females	1.32	0.76–2.28
Males	**1.85**	**1.08–3.16**

Bold font indicates statistical significance (*P*-value <.05).

When comparing the standardized incidence of selected cancer types between CD patients and the general population, no specific cancer type was statistically significantly different (Table 3).

**Table 3.** Standardized incidence rates (SIR) for most common cancer types in IBSEN patients.

ICD-10 Cancer type	Observed males	Observed Females	SIR males	95% CI	SIR females	95% CI	SIR all	95% CI
Total	20	17	1.35	0.82–2.00	1.09	0.64–1.67	1.22	0.86–1.64
C15–26 Digestive organs	2	3	0.68	0.08–1.89	0.97	0.20–2.35	0.83	0.27–1.70
C18–C21 Colorectal	1	2	0.57	0.01–2.09	0.99	0.12–2.76	0.79	0.16–1.91
C30–34, C38 Respiratory and intrathoracic organs	4	3	2.40	0.65–5.26	2.15	0.44–5.19	2.29	0.92–4.27
C43 Malignant melanoma	2	0	2.28	0.28–6.34	0	nc	1.08	0.13–3.01
C44 Non-melanoma skin cancer	3	1	3.85	0.79–9.28	1.17	0.03–4.32	2.45	0.67–5.37
C43–C44 Skin	5	1	3.02	0.98–6.18	0.55	0.01–2.02	1.72	0.63–2.27
C50 Breast	0	5	0	nc	1.36	0.44–2.79	1.36	0.44–2.78
C51–63 Genital organs	5	2	1.18	0.38–2.42	1.00	0.12–2.78		
C64–68 Urinary tract	2	1	1.40	0.17–3.91	1.46	0.04–5.37	1.42	0.29–3.42
C73 Thyroid gland	0	1	0	nc	4.24	0.11–15.64	3.09	0.08–11.41
C81–96 Hematologic cancer	2	0	1.46	0.18–4.08	0	nc	0.77	0.09–2.15

Lung cancer was the most frequent cancer type, and the SIR approached statistical significance (SIR = 2.29, 95% CI: 0.92–4.27). All patients who were diagnosed with lung cancer had a history of tobacco smoking.

There were 4 cases of NMSC, which is more than twice the expected number. However, the incidence was not significantly increased, compared to the general population (SIR = 2.45, 95% CI: 0.67–5.37).

The incidence of colorectal cancer was not different compared with the general population (SIR = 0.79, 95% CI: 0.16–1.91).

In patients with CD, the prescription of thiopurines was not associated with a higher risk of subsequent cancer (HR = 0.93, 95% CI: 0.48–1.80).

## Discussion

The overall risk for cancer was significantly increased in patients with CD compared to the general population, although our data did not reveal statistically significant increased incidence for any specific cancer type.

Male patients specifically were at a higher risk of cancer than matched controls.

The same trend had already been revealed after 20 years of follow-up, but because there were fewer cancer cases, the precision of the estimates was limited.

Even after 30 years of follow-up, the median age of patients with CD in the IBSEN cohort is still relatively low, so the expected incidence of cancer is correspondingly low. This underscores the need for long-term studies, as patients with Crohn’s disease are often diagnosed as young adults.

In spite of the small number of incident cancer cases in patients with CD in the IBSEN cohort, the observed increase in cancer risk corroborates with what has been found in comparable studies. Studies from nationwide and/or population-based Nordic countries which span several decades have also found that patients with CD are at an increased risk for cancer compared to the general population, to a similar magnitude, and more so than patients with ulcerative colitis (UC).^10–12^

A population-based cohort study from the Netherlands, including 1157 CD patients since 1991, also found an overall increased risk of cancer.^13^ In this cohort, the risks of NMSC and hematologic malignancies were particularly elevated, and were associated with exposure to immunosuppressive drugs and thiopurines.

In a nationwide Danish cohort study starting in 1978 with 13 756 patients, after 30 years of follow-up, patients with CD had an increased incidence of cancer compared to the general population, in particular, gastrointestinal cancer, hematologic malignancies, smoking-related cancer, and melanoma.^10^

Another long-term Danish cohort from Copenhagen with 373 patients, initiated in 1962, reported an increased risk of intestinal, respiratory, and skin cancers.^11^

In Finland, a nationwide register study over 20 years found that male patients with CD had an incidence of cancers higher than expected. This was essentially due to cancers of the small intestine, biliary tract cancers, NMSC, and myeloma.^12^

In the IBSEN cohort, the SIR of lung cancer was particularly increased and this finding is probably related to smoking, since all patients with lung cancers had a history of smoking. In the IBSEN cohort, the proportion of CD patients with a history of smoking at diagnosis was high in comparison with the general population, as is often the case for CD cohorts.^14,15^

Moreover, there were more cases of skin cancers than expected based on the estimates from the general Norwegian population. Treatment with thiopurines results in a higher risk of NMSC.^16^ Treatment with TNF-α inhibitors might be associated with melanoma though recent reviews do not support this association.^3,17^ In the IBSEN cohort, 1 patient with NMSC was prescribed thiopurines, 1 patient TNF-α inhibitor only, and 2 patients were prescribed both. Biologics were available approximately from the 10-year control visit, and their use was recorded until the 20-year visit. The analysis of dose–response is not possible either for biologics or for thiopurines.

Protection from UV rays and smoking cessation may help reduce the incidence of skin cancers and lung cancers.

Fewer hematologic malignancies than expected were diagnosed, contrary to what was observed in patients with UC from the same IBD cohort, particularly male patients. This is in spite of the higher proportion of CD patients exposed to thiopurines during the first 20 years compared to UC, and that thiopurines are known to increase the risk for hematological malignancies.^3^ The lower-than-expected incidence may be due to the fact that, within the IBSEN cohort, patients with CD are on average younger than patients with UC.

Few gastrointestinal or colorectal cancers were diagnosed, and the incidence was not significantly different from controls. In nationwide studies, patients with CD have been found at a high relative risk of small bowel cancer, especially when diagnosed before 17 years old.^18–20^ For example, a Norwegian and Swedish nationwide register study found the risk of small bowel adenocarcinoma in CD patients to be increased by 8 times compared to the general population.^18^ Because the proportion of patients diagnosed before 17 years was low, and small bowel cancer has an age-standardized incidence rate under 3 per 100 000 years in the Norwegian population, the IBSEN study is not sufficiently powered to assess the risk of small bowel cancer.

In the IBSEN cohort, young age at diagnosis has been associated with advanced disease and surgery.^21^ The high rate of intestinal surgery, including almost half of the patients, may have contributed to preventing gastrointestinal cancer development.^22^

A nationwide register-based cohort study in Denmark and Sweden, with patients included from 1969, found an increased risk of colorectal cancer diagnosis (HR = 1.40 95% CI: 1.27–1.53).^23^ The incidence of colorectal cancer in the general Norwegian population is high, which may diminish the estimated SIR of colorectal cancer in the present study.^24^

The SIR of breast cancer was not statistically significantly increased.

Lastly, in a previous study, after 30 years of follow-up, patients with CD of the IBSEN cohort did not have significantly higher mortality with cancer as the underlying cause of death.^25^

### Strengths and Limitations

The main strength of this prospective study is the well-defined inclusion criteria implemented by dedicated specialists and hospitals, designed to minimize selection bias, and the long-term follow-up. The population-based study design allows to include the diversity of CD presentation and clinical courses.

Norwegian national health registries and the national identity number allow data linkage across registries. In addition, the drawing of matched control individuals from the general population is made possible.

The CRN has very good data completeness for most cancer types and precisely characterized quality indicators.^26,27^

A limitation of our cohort is its moderate size, which inevitably limits the statistical power, most severely for rare events. The limited statistical power may have caused the observed difference in incidence of rare cancer types to appear under the significance level.

The long follow-up period partially compensated for the moderate cohort size by increasing the cumulative number of cancer cases.

No further prospective and systematic clinical data were collected after 20 years of follow-up; therefore, it was not possible to investigate possible associations between recent medical treatment or precise cumulative doses and cancer risk.

Relevant data for the controls, for example, known cancer risk factors such as smoking, were not available and consequently could not be adjusted for.

Basal cell carcinomas are not recorded by the CRN; therefore, comparing our results for NMSCs incidence to other cohorts which include this cancer type may be biased.^12,13^

## Conclusions

In our long-term study, we have estimated the current risk of cancer in a well-characterized population-based cohort of CD patients. The risk of cancer in CD patients of the IBSEN cohort was higher than for their matched controls, especially for males, which highlights the potential severity of CD and the risk of serious complications. The incidence of gastrointestinal cancers was not higher than expected.

## Data Availability

The data underlying this article cannot be shared publicly due to the restrictions imposed by the ethics committee, the public registers, and the national legal framework.

## References

[1] Torres J , Mehandru S, Colombel JF, Peyrin-Biroulet L. Crohn’s disease. Lancet. 2017;389(10080):1741–1755. 27914655 10.1016/S0140-6736(16)31711-1

[2] Lo B , Zhao M, Vind I, Burisch J. The risk of extraintestinal cancer in inflammatory bowel disease: a systematic review and meta-analysis of population-based cohort studies. Clin Gastroenterol Hepatol. 2021;19(6):1117–1138.e19. 32801010 10.1016/j.cgh.2020.08.015

[3] Gordon H , Biancone L, Fiorino G, et al. ECCO guidelines on inflammatory bowel disease and malignancies. J Crohns Colitis. 2022;17(6):827–854. 10.1093/ecco-jcc/jjac187 36528797

[4] Beaugerie L , Itzkowitz SH. Cancers complicating inflammatory bowel disease. N Engl J Med. 2015;372(15):1441–1452. 25853748 10.1056/NEJMra1403718

[5] Hovde O , Hoivik ML, Henriksen M, et al. Malignancies in patients with inflammatory bowel disease: results from 20 years of follow-up in the IBSEN study. J Crohns Colitis. 2017;11(5):jjw193–jjw577. 10.1093/ecco-jcc/jjw193 28453756

[6] Moum B , Ekbom A, Vatn MH, et al. Inflammatory bowel disease: re-evaluation of the diagnosis in a prospective population based study in south eastern Norway. Gut. 1997;40(3):328–332. 9135520 10.1136/gut.40.3.328 PMC1027081

[7] Solberg IC , Vatn MH, Hoie O, et al. Clinical course in Crohn’s disease: results of a Norwegian population-based ten-year follow-up study. Clin Gastroenterol Hepatol. 2007;5(12):1430–1438. 18054751 10.1016/j.cgh.2007.09.002

[8] Adamo M , Groves C, Dickie L, Ruhl J. SEER Program Coding and Staging Manual 2023. National Cancer Institute, Bethesda, MD 20892. U.S. Department of Health and Human Services National Institutes of Health National Cancer Institute.

[9] Numbers from the Cancer registry of Norway. https://sb.kreftregisteret.no/. Accessed June 2022.

[10] Kappelman MD , Farkas DK, Long MD, et al. Risk of cancer in patients with inflammatory bowel diseases: a nationwide population-based cohort study with 30 years of follow-up evaluation. Clin Gastroenterol Hepatol. 2014;12(2):265–273.e1. 23602821 10.1016/j.cgh.2013.03.034 PMC4361949

[11] Burisch J , Lophaven S, Langholz E, Munkholm P. The clinical course of Crohn’s disease in a Danish population-based inception cohort with more than 50 years of follow-up, 1962-2017. Aliment Pharmacol Ther. 2022;55(1):73–82. 34543457 10.1111/apt.16615

[12] Jussila A , Virta LJ, Pukkala E, Farkkila MA. Malignancies in patients with inflammatory bowel disease: a nationwide register study in Finland. Scand J Gastroenterol. 2013;48(12):1405–1413. 24131389 10.3109/00365521.2013.846402

[13] van den Heuvel TR , Wintjens DS, Jeuring SF, et al. Inflammatory bowel disease, cancer and medication: Cancer risk in the Dutch population-based IBDSL cohort. Int J Cancer. 2016;139(6):1270–1280. 27170593 10.1002/ijc.30183

[14] Maaser C , Langholz E, Gordon H, et al. European Crohn’s and Colitis Organisation topical review on environmental factors in IBD. J Crohns Colitis. 2017;11(8):905–920. 28039310 10.1093/ecco-jcc/jjw223

[15] Statistics Norway. Percentage Daily Smokers and Occasional Smokers, by Sex and Age (Per Cent) 1973–2021. 2022. https://www.ssb.no/en/statbank/table/05307. Accessed June 2022.

[16] Solitano V , D’Amico F, Correale C, Peyrin-Biroulet L, Danese S. Thiopurines and non-melanoma skin cancer: partners in crime in inflammatory bowel diseases. Br Med Bull. 2020;136(1):107–117. 33200781 10.1093/bmb/ldaa033

[17] Esse S , Mason KJ, Green AC, Warren RB. Melanoma risk in patients treated with biologic therapy for common inflammatory diseases: a systematic review and meta-analysis. JAMA Dermatol. 2020;156(7):787–794. 32432649 10.1001/jamadermatol.2020.1300 PMC7240639

[18] Yu J , Refsum E, Perrin V, et al. Inflammatory bowel disease and risk of adenocarcinoma and neuroendocrine tumors in the small bowel. Ann Oncol. 2022;33(6):649–656. 35276334 10.1016/j.annonc.2022.02.226

[19] Elmahdi R , Lemser CE, Thomsen SB, et al. Development of cancer among patients with pediatric-onset inflammatory bowel disease: a meta-analysis of population-based studies. JAMA Netw Open. 2022;5(3):e220595. 35230438 10.1001/jamanetworkopen.2022.0595 PMC8889462

[20] Axelrad JE , Olen O, Sachs MC, et al. Inflammatory bowel disease and risk of small bowel cancer: a binational population-based cohort study from Denmark and Sweden. Gut. 2021;70(2): gu2020. 10.1136/gutjnl-2020-320945 32474410

[21] Solberg IC , Cvancarova M, Vatn MH, Moum B, Group IS. Risk matrix for prediction of advanced disease in a population-based study of patients with Crohn’s disease (the IBSEN Study). Inflamm Bowel Dis. 2014;20(1):60–68. 24280875 10.1097/01.MIB.0000436956.78220.67

[22] Lunder AK , Jahnsen J, Bakstad LT, et al. Bowel damage in patients with long-term Crohn’s disease, assessed by magnetic resonance Enterography and the Lemann index. Clin Gastroenterol Hepatol. 2018;16(1):75–82 e75. 28694130 10.1016/j.cgh.2017.06.053

[23] Olen O , Erichsen R, Sachs MC, et al. Colorectal cancer in Crohn’s disease: a Scandinavian population-based cohort study. Lancet Gastroenterol Hepatol. 2020;5(5):475–484. 32066530 10.1016/S2468-1253(20)30005-4

[24] Arnold M , Sierra MS, Laversanne M, et al. Global patterns and trends in colorectal cancer incidence and mortality. Gut. 2017;66(4):683–691. 26818619 10.1136/gutjnl-2015-310912

[25] Follin-Arbelet B , Cvancarova Smastuen M, Hovde O, Jelsness-Jorgensen LP, Moum B. Mortality in patients with inflammatory bowel disease: results from 30 years of follow-up in a Norwegian Inception Cohort (the IBSEN study). J Crohns Colitis. 2023;17(4):497–503. 36239614 10.1093/ecco-jcc/jjac156 PMC10115228

[26] Larsen IK , Smastuen M, Johannesen TB, et al. Data quality at the Cancer Registry of Norway: an overview of comparability, completeness, validity and timeliness. Eur J Cancer. 2009;45(7):1218–1231. 19091545 10.1016/j.ejca.2008.10.037

[27] Bakken IJ , Gystad SO, Christensen OO, et al. Comparison of data from the Norwegian Patient Register and the Cancer Registry of Norway. Tidsskr Nor Laegeforen. 2012;132(11):1336–1340. 22717858 10.4045/tidsskr.11.1099

